# Purified cell wall from the probiotic bacterium *Lactobacillus gasseri* activates systemic inflammation and, at higher doses, produces lethality in a rat model

**DOI:** 10.1186/cc13966

**Published:** 2014-07-02

**Authors:** Xinhui Xu, Caitlin Hicks, Yan Li, Junwu Su, Joseph Shiloach, Jeanne B Kaufman, Yvonne Fitz, Peter Q Eichacker, Xizhong Cui

**Affiliations:** 1 Critical Care Medicine Department, Clinical Center, National Institutes of Health, Bethesda, MD 20892, USA; 2 NIDDK, National Institutes of Health, Bethesda, MD 20892, USA; 3 Critical Care Medicine Department, Renji Hospital, School of Medicine, Shanghai Jiaotong University, Shanghai, China

## Abstract

**Introduction:**

One proposed benefit of probiotic therapy is that probiotic bacterial cell-wall binding to intestinal cell pathogen-recognition receptors activates protective innate immunity. However, in critically ill patients, intestinal epithelium disruption by shock or other insults may compromise this compartmentalized response and cause systemic bacteria and cell-wall translocation. The effects of intravascular introduction of probiotic bacterial cell wall are unclear.

**Methods:**

We investigated 24-hour infusions of purified cell wall from *Lactobacillus gasseri* ATC33323 (*L. gasseri*), a probiotic bacterium, in Sprague–Dawley rats (*n* = 49).

**Results:**

Increasing cell-wall doses (0 (control), 10, 20, 40, 80, or 160 mg/kg over 24 hours) produced dose-ordered decreases in survival measured after 168 hours (11 survivors/11 total (100%), seven of seven (100%), seven of seven (100%), six of eight (75%), five of eight (63%), and one of nine (11%), respectively, *P* < 0.0001). The *L. gasseri* cell wall was equally or more lethal than *Staphylococcus aureus* cell wall, which was previously studied (100% to 88% survival with the same increasing doses). During challenge, compared with controls, *L. gasseri* cell wall produced increases in blood IL-1β, IL-10, tumor necrosis factor-α, migratory inhibitory protein-1α, monocyte chemotactic protein-1, and nitric oxide, and decreases in neutrophils, lymphocytes, and platelets that were greater with higher versus lower doses (*P* ≤ 0.05). Medium-dose cell wall (40 and 80 mg/kg combined) progressively decreased blood pressure and increased heart rate, and all doses increased lactate, hepatic transaminases, and creatinine phosphokinase (*P* ≤ 0.05).

**Conclusion:**

Although *L. gasseri*, like other probiotic bacteria, is considered safe, its cell wall can stimulate the maladaptive inflammatory response associated with pathogenic bacteria. Such effects deserve study, especially regarding critically ill patients.

## Introduction

Probiotic therapy to improve intestinal barrier and immune function and reduce growth and translocation of pathogenic bacteria is increasing and has been applied in critically ill patients for several conditions [1-5]. Many probiotic preparations include *Lactobacillus* species that are thought to be of little clinical risk [4]. Despite their widespread use however, concerns over probiotics, including ones with *Lactobacillus* strains, have been raised [6-15]. Although the bacteria used in probiotic preparations appear nonpathogenic, if they were to grow to large numbers in the intestine of a critically ill patient in whom mucosal integrity was compromised, translocation of the bacteria or their biologically active components into the systemic circulation could occur. In sufficient concentrations, such translocation could activate a systemic inflammatory response [6-8,11-15]. The cell wall of *Lactobacillus* strains includes peptidoglycan and other components that bind to pathogen-recognition receptors (PRRs) on host cells [5,16-20]. Although one of the proposed attributes of probiotic *Lactobacillus* strains is that this binding in intestinal tissue stimulates protective innate immune responses [16,21], if it is not adequately compartmentalized, such stimulation systemically could be deleterious. At present however, no data describe the potential effects of intravascular introduction of *Lactobacillus* cell wall. We therefore investigated the effects of 24-hour infusions of purified cell wall from *Lactobacillus gasseri* ATC33323 (*L. gasseri*), a probiotic bacterium [5,22], in a rat model. The range and method of administration of *L. gasseri* cell-wall doses, as well as the rat model used, were similar to those in prior studies testing *Staphylococcus aureus* and *Bacillus anthracis* cell walls [23].

## Materials and methods

### Animal care

This study protocol was approved by the Animal Care and Use Committee of the Clinical Center of the National Institutes of Health (Animal Study Protocol CCM 0601).

### Study design

In weekly experiments, Sprague–Dawley rats (*n* = 49 total) with carotid arterial and jugular venous catheters were randomized to receive 24-hour intravenous infusions (0.5 ml/h) of *L. gasseri* cell wall in total doses of 10, 20, 40, 80, or 160 mg/kg or of diluent only (control). Mean arterial blood pressures (MBPs) and heart rates (HRs) were measured immediately before and at 1 hour intervals from 1 to 10 hours and at 2-hour intervals from 10 to 24 hours after initiation of infusion. Arterial blood was collected at 4, 8, and 24 hours for blood gas (ABG), lactate, complete blood cell (CBC), cytokine, nitric oxide (NO), alanine and aspartate aminotransferases (ALT and AST, respectively), creatine phosphokinase (CPK), blood urea nitrogen (BUN), and creatinine measures. Animals had similar volumes (0.5 ml) of blood drawn and normal saline replaced at each time point, Survival was assessed at 168 hours.

### Cell-wall preparations

The *L. gasseri* ATC3323 were obtained from Dr. T. R. Klaenhammer, Department of Food, Bioprocessing, and Nutrition Sciences, North Carolina State University, Raleigh, NC, USA. Cell wall was purified by using previously described Methods [23]. In brief, bacteria grown to the late exponential phase were harvested with centrifugation, washed in distilled water (endotoxin-free), and boiled in an equal volume of 8% sodium dodecylsulfate (SDS) for 30 minutes. After incubation overnight at room temperature with agitation, the suspension was centrifuged, and the pellet extracted twice by boiling with 4% SDS. The extract was then washed, centrifuged at 20°C, and diluted with 1× phosphate-buffered saline to deliver doses of 10, 20, 40, 80, and 160 mg/kg body weight when administered as an infusion over 24 hours in a total volume of 12 ml (0.5 ml/h).

Agarose gel electrophoresis with ethidium bromide staining and SDS-PAGE with coomassie blue staining did not detect DNA/RNA or protein contamination in purified *L. gasseri* cell wall. As determined by the chromogenic limulus amoebocyte lysate assay (Clonogen, Germantown, MD, USA), the lipopolysaccharide (LPS) content of the cell-wall preparation was 0.05 ng/mg. Based on the average size of animals studied, the maximum LPS amount administered during a 24-hour cell-wall infusion would have been 8 ng/kg. As reported in prior cell-wall studies, to confirm that LPS contamination at a level of 0.05 ng/mg would not confound cell-wall effects on hemodynamic, arterial blood gas, circulating cell, cytokine, or NO measures, rats (*n* = 6) were challenged with a total dose of LPS comparable to what would be received if the highest cell-wall dose tested (160 mg/kg) were contaminated with LPS at a level of 0.05 ng/mg cell wall (that is, 8 ng/kg infused over a 24–hour period). This dose of LPS produced no lethality and, compared with diluent control, did not significantly alter any of the parameters investigated in the present study [23].

### Laboratory measurements

Arterial blood pressure, HR, ABG, lactate, and CBC measures and samples for cytokine and NO levels were obtained as previously described [23,24]. Cytokines (interleukin-1β (IL-1β), IL-2, IL-6, IL-10, tumor necrosis factor-α (TNF-α), granulocyte-macrophage colony-stimulating factor (GM-CSF), monocyte chemotactic protein-1 (MCP-1), migratory inhibitory protein-1α (MIP-1α), and regulated on activation, normal T-cell expressed and secreted (RANTES)) were measured by using a standard kit (Cytokine Multiplex Immunoassay Kit, Millipore, Danvers, MA, USA). Plasma nitrite/nitrate (NO) levels were measured by using a fluorometric assay kit (Cayman Chemical, Ann Arbor, MI, USA). Chemistry analysis was conducted with the Drew Trilogy Analyzer (Diamond Diagnostics, Holliston, MA, USA).

### Statistics

Statistical Analysis System Version 9.3 software (SAS Institute, Inc, Cary, NC, USA) was used for all the analysis. Kaplan-Meier survival curves were used to show survival effects, and a Wilcoxon rank test was used in PROC LIFETEST to compare the effect of cell-wall doses on survival. All other parameters were analyzed with two-way ANOVA accounting for cell wall dose (each dose versus control or between doses) and time point of observation, and one-way ANOVA to compare the effect of the cell wall with control at each time point. For clarity in figures, serial effects of each dose of cell wall (that is, cell wall minus control) are shown. Logarithmic transformation was used when necessary. Two-sided *P* values of less than 0.05 were considered significant. Multiple comparisons were not adjusted for.

## Results

### Survival

**Figure 1:**
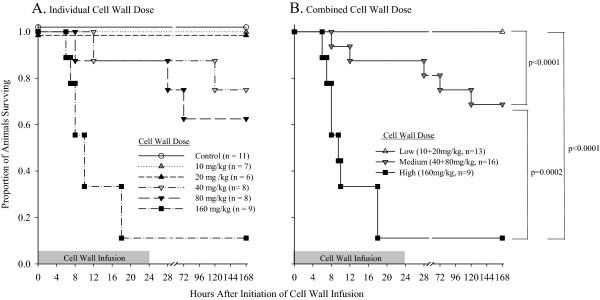
**Comparison of survival. (A)** Number of animals randomized to be challenged with diluent alone (control) or to one of five increasing *L. gasseri* cell-wall doses, including 10, 20, 40, 80, or 160 mg/kg (administered over a 24-hour period as a continuous infusion) and the proportion of animals from each group surviving over time. Survival did not differ comparing the 10 versus 20 mg/kg *L. gasseri* cell-wall doses (low doses) or the 40 versus 80 m/kg doses (medium doses), and these were combined for subsequent analysis. **(B)** The proportion of animals surviving over time for the low (10 and 20 mg/kg doses combined) versus medium (40 and 80 mg/kg doses combined) versus high (160 mg/kg) dose cell-wall groups.

All animals challenged with diluent alone (controls, *n* = 11) survived (Figure 1A). Challenge with increasing doses of *L. gasseri* cell wall (10, 20, 40, 80, or 160 mg/kg) produced decreasing survival (seven survived of seven studied (100%), seven of seven (100%), six of eight (75%), five of eight (63%), and one of nine (11%), respectively) (*P* < 0.0001 for the effect of increasing cell-wall dose on decreasing survival). However, survival did not differ significantly comparing either the 10 versus 20 mg/kg or 40 versus 80 m/kg *L. gasseri* cell-wall doses. These groups were combined to increase the power to detect the influence of cell wall and for presentation of data described later (see Methods also). Overall, survival was significantly different, comparing the low (10 and 20 mg/kg doses combined) versus medium (40 and 80 mg/kg doses combined) versus high (160 mg/kg) cell-wall doses (*P* ≤ 0.0002) (Figure 1B).

### Inflammatory cytokine (log (pg/ml)), nitric oxide (μ*M*), and neutrophil, lymphocyte, and platelet levels (all × 10^3^ cells/μl)

**Figure 2:**
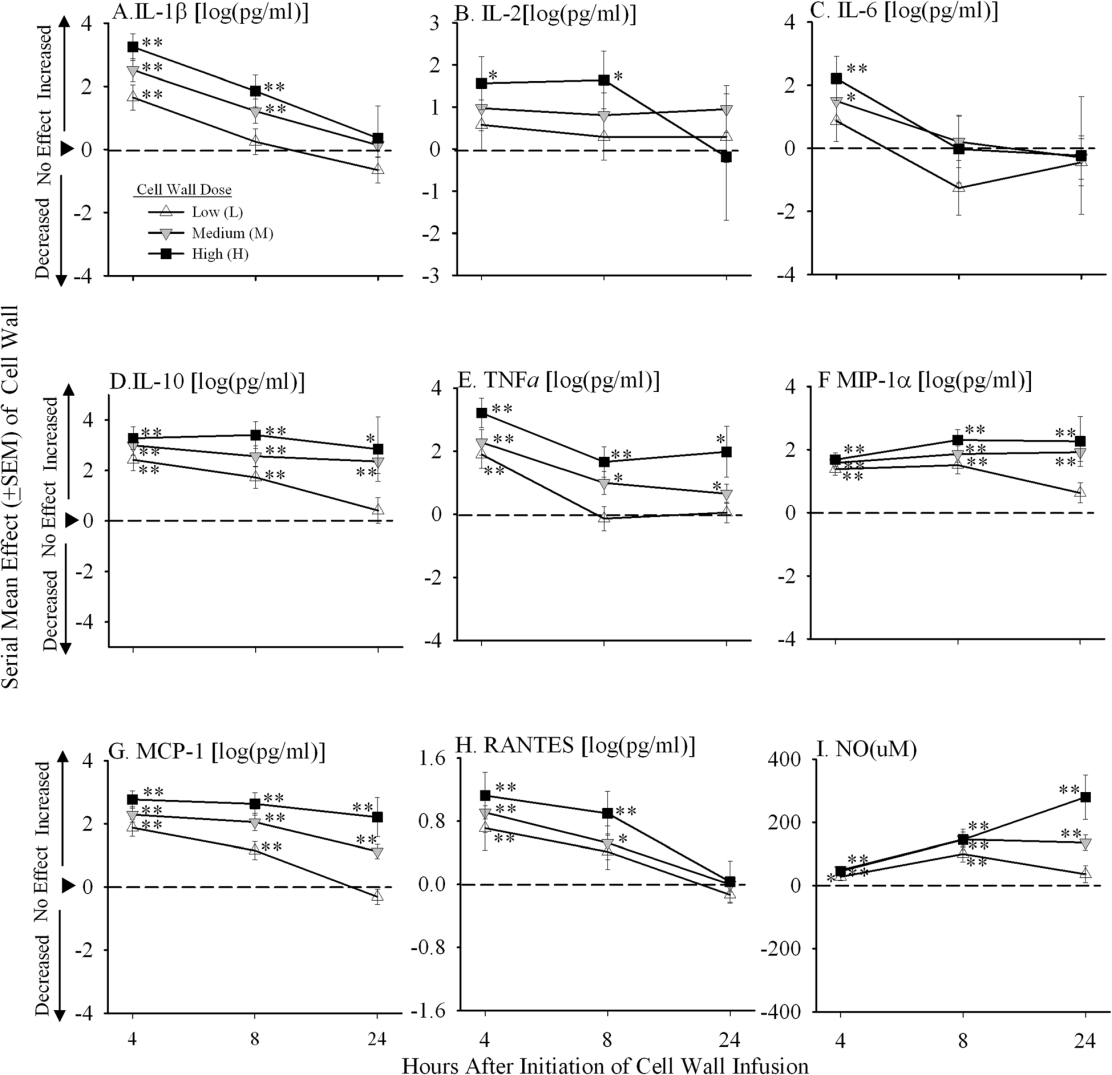
**Serial mean effects (±SEM) of low (L), medium (M), or high (H) cell-wall doses compared with controls (phosphate-buffered saline; see Methods regarding calculation of the effects) on log IL-1β, log IL-2, log IL-6, log IL-10, log TNF-α, log MIP-1α, log MCP-1, log RANTES, and NO levels at 4, 8, and 24 hours after the initiation of challenges (designated on the x axis as 4, 8, or 24).** Significant effects are designated by asterisks (* ≤ 0.05; ** ≤ 0.0001).

Compared with controls, at 4 hours after the start of the 24-hour challenge, low, medium, and high doses of *L. gasseri* cell wall each increased (mean effect of cell wall versus control, ±SEM)) IL-1β (1.65 ± 0.40, 2.52 ± 0.36, 3.24 ± 0.42) , IL-10 (2.42 ± 0.43, 2.99 ± 0.39, 3.28 ± 0.45), TNF-α (1.90 ± 0.44, 2.28 ± 0.40, 3.21 ± 0.47), MIP-1α (1.38 ± 0.20, 1.58 ± 0.18, 1.68 ± 0.21), MCP-1 (1.87 ± 0.27, 2.29 ± 0.24, 2.77 ± 0.28), RANTES (0.71 ± 0.28, 0.91 ± 0.26, 1.12 ± 0.30), and NO (28.0 ± 11.9, 48.8 ± 11.2, 45.7 ± 13.6) (all *P* ≤ 0.05). Medium and high doses increased IL-6 (1.50 ± 0.60, 2.21 ± 0.70) (both *P* ≤ 0.05); and high dose increased IL-2 (1.56 ± 0.63) and GM-CSF (1.24 ± 0.50) (both *P* ≤ 0.05) (Figure 2, data for GM-CSF not shown). These increases persisted with some cell-wall doses while decreasing at 8 and/or 24 hours with others (see Figure 2). Overall, higher cell-wall doses had greater effects than lower ones in patterns that were significant either at all time points for IL-1β, IL-10, TNF-α, GM-CSF, and MCP-1 (*P* ≤ 0.0004 for high versus medium versus low-dose cell wall) or at later ones for MIP-1α and NO (*P* ≤ 0.003 for the interaction with time).

**Figure 3:**
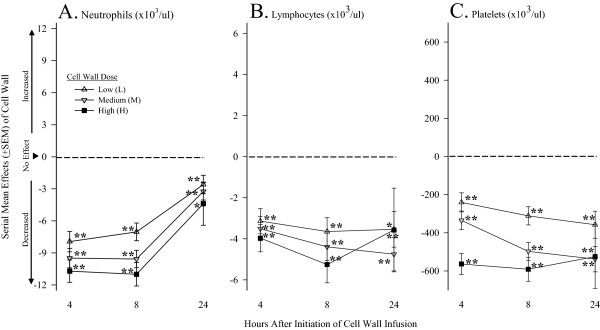
**Serial mean effects (±SEM) of low (L), medium (M), or high (H) cell-wall doses compared with controls (phosphate-buffered saline; see Methods regarding calculation of the effects) on circulating neutrophil, lymphocyte, and platelet concentrations at 4, 8, and 24 hours after the initiation of challenges (designated on the x axis as 4, 8, or 24).** Significant effects are designated by asterisks (* ≤ 0.05; ** ≤ 0.0001).

Compared with controls, at 4 hours, low, medium, and high cell-wall doses each respectively decreased circulating neutrophils (-7.94 ± 0.95, -9.5 ± 0.91, and -10.71 ± 1.04), lymphocytes (-3.214 ± 0.61, -3.51 ± 0.58, and -3.97 ± 0.67) and platelets (-241 ± 50, -335 ± 48, and -563 ± 55) (all *P* ≤ 0.05) (Figure 3). These decreases persisted for all cell types with each of the three cell-wall doses. However, decreases in neutrophils were not so great later with low and medium cell-wall doses, and decreases in platelets were greater later with medium doses (*P* ≤ 0.03 for the time interactions). Overall, higher cell-wall doses had greater effects than lower ones in patterns that were significant for neutrophils and platelets (*P* < 0.0001 for the effects of high versus medium versus low-dose cell wall) and approached significance for lymphocytes (*P* = 0.056).

### Mean arterial blood pressure (MBP, mm Hg) and heart rate (HR, BPM) measurements

**Figure 4:**
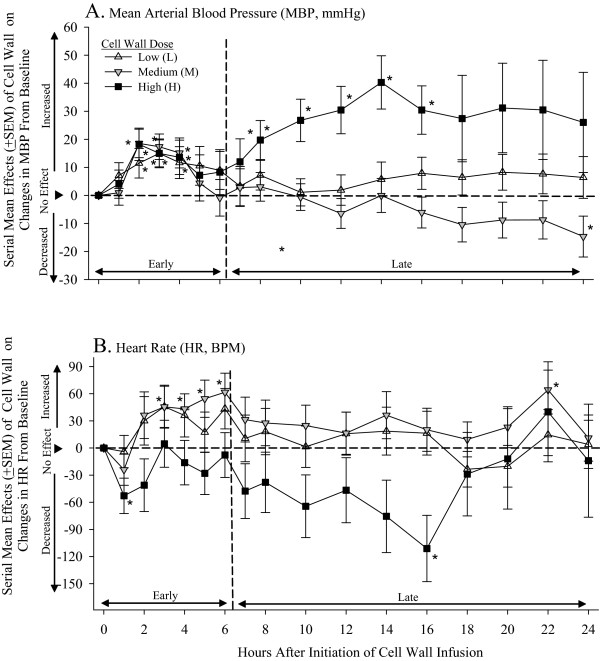
**Mean effects (±SEM) of low (L), medium (M), or high (H) cell-wall doses compared with controls (phosphate-buffered saline, see Methods regarding calculation of the effects) on serial changes in mean arterial blood pressure (MBP, mm Hg), and heart rate (HR, BPM) in all animals during the 24-hour challenges.** As described in the Results section, to analyze these changes, data were divided into the 6-hour period of challenge before the onset of lethality (early period) and the subsequent 18-hour period (later period). Significant effects are designated by the asterisks (* ≤ 0.05; ** ≤ 0.0001). Compared with controls, low-dose cell wall increased MBP early and later, and HR, early (*P* ≤ 0.003 averaged over time). Medium dose increased and then decreased MBP and HR early (*P* ≤ 0.03 for the time interaction), and then decreased MBP and increased HR later (*P* ≤ 0.0007 averaged over time). High dose paradoxically increased MBP and decreased HR both early and later (*P* ≤ 0.02 averaged over time).

To analyze the effects of *L. gasseri* cell wall on changes in MBP and HR during the 24-hour challenges, data were divided into the 6-hour period before the onset of lethality (early period) and the subsequent 18-hour period (later period) during which lethality was observed in the medium- and high-dose groups (Figure 4). Compared with controls, low-dose cell wall significantly increased MBP early and later, and HR early (*P* ≤ 0.003 averaged over time). Medium dose increased and then decreased MBP and HR early (*P* ≤ 0.03 for the time interaction), and then decreased MBP and increased HR later (*P* ≤ 0.0007 averaged over time). High dose paradoxically increased MBP and decreased HR both early and later (*P* ≤ 0.02 averaged over time). Although the effects of the three cell-wall doses on MBP did not differ significantly early, their effects on MBP later and on HR both early and later did (*P* < 0.0001).

### Lactate, bicarbonate, and base excess, and AST, ALT, and CPK

**Figure 5:**
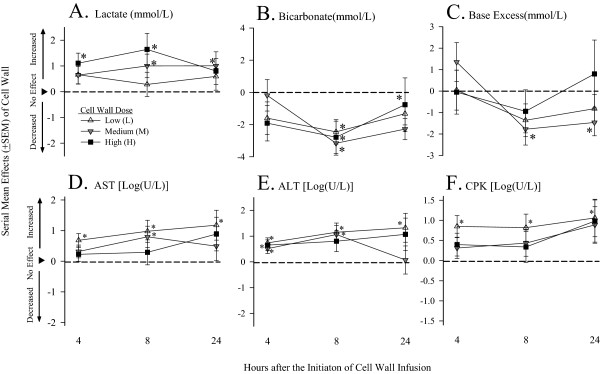
**Serial mean effects (±SEM) of low (L), medium (M), or high (H) cell-wall doses compared with controls (phosphate-buffered saline; see Methods regarding calculation of the effects) on lactate, bicarbonate, base excess, aspartate and alanine aminotransaminases (AST and ALT, respectively), and creatine phosphokinase (CPK) at 4, 8, and 24 hours after the initiation of challenges (designated on the x axis as 4, 8, or 24).** Significant effects are designated by the asterisks (* ≤ 0.05; ** ≤ 0.0001).

Compared with controls at 8 hours, low-dose cell wall decreased bicarbonate (-2.45 ± 0.76). At 8 and 24 hours, medium dose increased lactate (1.01 ± 0.45 and 1.01 ± 0.29) and decreased bicarbonate (-3.16 ± 0.73 and -2.28 ± 0.65) and base excess (*M*; -1.78 ± 0.73 and -1.46 ± 0.62). At 4 and 8 hours, high dose increased lactate (1.11 ± 0.39 and 1.65 ± 0.62), and at 8 hours, decreased bicarbonate (-2.79 ± 1.00) (all *P* ≤ 0.03) (Figure 5). The effects of the three *L. gasseri* cell-wall doses on lactate, bicarbonate, and base excess did not differ significantly.

Compared with controls, at 4, 8, and 24 hours, low-dose cell wall increased AST (Log (U/L) 0.68 ± 0.21, 0.98 ± 0.35, and 1.18 ± 0.49, respectively), ALT (0.74 ± 0.19, 1.15 ± 0.35, and 1.32 ± 0.56), and CPK (0.85 ± 0.26, 0.82 ± 0.33, and 1.06 ± 0.47) (all *P* ≤ 0.03). At 8 hours, medium-dose cell wall increased AST (0.80 ± 0.35), and at 4 and 8 hours, increased ALT (0.52 ± 0.18 and 1.06 ± 0.34) (all *P* ≤ 0.02). At 4 hours, high-dose cell wall increased ALT (0.65 ± 0.20) (*P* = 0.003) (Figure 5). The effects of cell wall on AST, ALT, and CPK did not differ significantly comparing the low, medium, and high doses.

## Discussion

The 24-hour infusions with increasing *L. gasseri* cell-wall doses increased inflammatory cytokine and NO levels and decreased circulating neutrophils, lymphocytes, and platelets. Increases in intravascular inflammatory cytokines likely caused vascular endothelial activation and adherence of circulating leukocytes and platelets [25]. These changes were all greater with higher versus lower doses and show that the *L. gasseri* cell wall can stimulate a robust intravascular inflammatory response. Elements in this type of response contribute to the septic shock and organ injury occurring with cell wall from gram-positive bacteria known to be pathogenic for humans, such as *Staphylococcus aureus*[20]. Consistent with pathogenic bacteria, *L. gasseri* cell wall produced reductions in survival that were dose dependent, hypotension, and tachycardia with medium doses, and evidence of tissue hypoperfusion with all doses, manifested by increases in lactate, hepatic enzymes, and creatine phosphokinase levels. Notably, the effects of *L. gasseri* cell wall on survival and changes in inflammatory cytokines, NO, and circulating leukocytes and platelets in the present study occurred in patterns very similar to ones observed with the same doses of purified *S. aureus* and *Bacillus anthracis* cell wall in a prior study with the same rat model [23].

Several lines of evidence support the inflammatory effects seen with *L. gasseri* cell wall in this rat model. Whole *L. gasseri* bacteria or cell-wall extracts alone stimulated inflammatory cytokine production, including IL-1β, IL-6, IL-10, TNF-α, MCP-1, MIP-1α, or GM-CSF, or NO levels from either murine J774.1 or RAW264.7 macrophages or human myeloid dendritic cells [16,21]. Heat-killed *L. gasseri* administered orally in Balb/c mice activated splenic natural killer cells and increased pulmonary inflammatory cytokine (TNF-α, INF-γ, and IL-12) mRNA expression [21,26]. In this latter study, immune stimulation by *L. gasseri* was actually protective during influenza viral infection [26]. This may have been because *L. gasseri* remained compartmentalized in the intestinal space. The findings from the present study raise the possibility, however, that if compartmentalization is disrupted and *L. gasseri* or its components translocate to the intravascular space, they could elicit a maladaptive inflammatory response.

How *L. gasseri* cell wall elicits an inflammatory response is unclear. Few data exist regarding this cell wall’s precise structure. However, earlier work showed that *L. gasseri* cell wall consists of at least three components, including peptidoglycan, a neutral polysaccharide, and an anionic polysaccharide [27]. Structural aspects of the peptidoglycan component were very similar to peptidoglycan in the cell wall of *S. aureus*[28]. Binding of peptidoglycan from *S. aureus* to the PRR Toll-like receptor 2 (TRL2) is believed to play an important role in the pathogenesis of the injurious inflammatory response with which these bacteria are associated [29]. It is, therefore, noteworthy that stimulation of TNF-α production from human myeloid dendritic cells by live *L. gasseri* has been shown also to be mediated in part by TLR2 [30]. Of note as well, the neutral polysaccharide found in the *L. gasseri* cell wall is similar to one found in *S. pneumococcus*, another pathogenic gram-positive bacterium [27]. 

Interestingly, the highest dose of *L. gasseri* cell wall in the present study appeared to have greater lethal effects than comparable doses of *S. aureus* or *B. anthracis* in the prior studies noted [23]. These differences are difficult to interpret because the subject animals were derived from differing batches and were investigated during different time periods. However, while the peptidoglycan of these three bacteria may be relatively similar, it is likely that differences in the teichoic acids and glycopolymers making up the cell wall of each could produce differing physiologic responses [31].

The clinical implications of the present findings are not clear. It required 1 × 10^10^ CFU of *L. gasseri* to produce 1 mg of cell wall for these experiments. Thus, lethal cell-wall doses (40 to 160 mg/kg) were equivalent to infection with 10 to 40 × 10^10^ CFU/kg bw or 6 to 24 × 10^9^ CFU/ml blood (based on the estimated blood volume of a rat weighing 250 g). These concentrations are comparable to those in a study of *L. casei*, a bacterium commonly used in probiotics, reporting that the 50% lethal dose in mice was 9 × 10^9^ CFU/kg bw, whereas the minimal lethal dose in rats was 40 × 10^9^ CFU/kg bw [32].

However, it is difficult to compare these bacterial doses with the probiotic doses used clinically. Only one report has provided semiquantitative blood bacteria counts from patients with probiotic-related sepsis, and this study did not quantify counts greater than 100 cfu/ml [12]. The development of sepsis during probiotic use in patients has actually appeared more related to the degree of underlying illness than to the dose of probiotic itself [10,12]. Although daily doses of 2.5 × 10^11^ CFU/kg for up to a year and single doses of up to 10^13^ CFU/kg have been well tolerated in healthy patients [10], doses of only 10^10^ CFU in severely ill patients have resulted in sepsis and bacteremia [7,9,12,33,34].

It is possible that the concentrations of *L. gasseri* cell wall eliciting the responses noted in the present study are rarely if ever reached clinically. A rationale underlying the bacterial strains used in probiotics is that they have a high affinity for binding to intestinal epithelium and that they are minimally invasive [4,5]. Conversely, however, the finding that *L. gasseri* cell wall can elicit an intravascular inflammatory response comparable to that of bacteria such as *S. aureus*[23], a known human pathogen, must raise concern clinically.

In a review of 241 *Lactobacillus* infections in patients, bacteremia was noted in 129 cases, and the overall mortality rate was 29.1% [35]. Caution would appear especially warranted in critically ill patients in whom intestinal integrity may be disrupted [7,10,12,33,36]. Whether effects such as the ones noted in the present study relate to adverse effects noted with probiotic therapies clinically is not clear but would appear to warrant further consideration [7,35,37]. It is important to point out however, that the therapeutic effects of probiotics such as those *L. gasseri* is included in, rely on whole living bacteria. Also, to identify an agent as a probiotic requires that it be alive. The cell wall tested here was derived from killed bacteria, and it does not represent the entire bacterium.

This study has potential limitations. First, although the present model used 24-hour cell-wall infusions to permit the gradual systemic introduction of bacterial products, it does not reproduce the early pathogenesis of translocation. Thus, introduction of cell wall during the natural process of translocation may elicit a very different host response from the one observed here.

Second, although whole cell wall was used for study, it is likely that, as for other gram-positive bacteria, much of the preparation’s effects were related to the peptidoglycan component [20,38-40]. Further investigating the individual components making up the *L. gasseri* cell wall would be informative.

Third, the highest dose of *L. gasseri* cell wall studied had paradoxic effects on MBP and HR, actually increasing the former and decreasing the latter. The basis for this response is unknown, although it might be related to a primary central nervous system effect of this cell-wall dose. Despite these paradoxic effects with the most lethal dose, lethality with the medium cell-wall dose was associated with progressive reductions in blood pressure and increases in heart rate, as would be expected in a state of sepsis.

Fourth, animal numbers may appear relatively small in the study groups. However, even with these numbers, differences in survival, the primary outcome comparing the low, medium, and high cell-wall doses, were highly significant. With such differences, it would not have been possible, from an animal care and use perspective, to justify further animals for study. Finally, multiple comparisons were not adjusted for.

## Conclusions

*Lactobacillus gasseri* is considered to be a relatively safe bacterium and, like other *Lactobacillus* strains, is commonly included in probiotic preparations [5]. However, the present study demonstrated that its cell wall is capable of stimulating the type of maladaptive inflammatory response typically associated with far more pathogenic gram-positive bacteria. Such effects may deserve further study, especially with regard to the use of probiotics, including strains like *L. gasseri*, in critically ill patients.

## Key messages

• Purified cell wall from *L. gasseri,* a strain of bacteria considered safe and included in probiotic preparations used in critically ill patients, when introduced into the systemic circulation of rats, produced a robust inflammatory response and lethality, comparable to the effects of cell wall from *S. aureus*.

• Although stimulation of innate immunity is thought to be a key benefit of bacteria used in probiotic preparations, better understanding the effects of these bacteria introduced systemically may be important, especially for preparations used in critically ill patients with compromised gut integrity.

## Abbreviations

ABG: Arterial blood gas; ALT: alanine aminotransferases; ANOVA: analysis of variance; AST: aspartate aminotransferase; *B. anthracis*: *Bacillus anthracis*; BUN: blood urea nitrogen; CBC: complete blood cell; CPK: creatine phosphokinase; GM-CSF: granulocyte-macrophage colony-stimulating factor; HR: heart rate; IL-10: interleukin-10; IL-1β: interleukin-1β; IL-2: interleukin-2; IL-6: interleukin-6; *L. gasseri*: *Lactobacillus gasseri*; LPS: lipopolysaccharide; MABP: mean arterial blood pressure; MCP-1: monocyte chemotactic protein-1; MIP-1α: migratory inhibitory protein-1α; NO: nitric oxide; PRR: pathogen recognition receptors; RANTES: regulated on activation, normal T-cell expressed and secreted; *S. aureus*: *Staphylococcus aureus*; SDS: sodium dodecylsulfate; TNF-α: tumor necrosis factor-α; TRL2: Toll-like receptor 2.

## Competing interests

All authors declare no conflicts of interest.

## Authors' contributions

XX conceived the study design, collected and analyzed the data, and wrote the manuscript. CH, YL, JSu, and YF contributed substantially to collecting and interpreting the data. JSh and JBK produced the cell wall and contributed to manuscript preparation. XC and PQE conceived the study design, analyzed and interpreted the data, and wrote the manuscript. All authors read and approved the final manuscript.
